# Identification of the KCNQ1OT1/ miR-378a-3p/ RBMS1 Axis as a Novel Prognostic Biomarker Associated With Immune Cell Infiltration in Gastric Cancer

**DOI:** 10.3389/fgene.2022.928754

**Published:** 2022-07-14

**Authors:** Ting Yue, Jingjing Li, Manguang Liang, Jiaman Yang, Zhiwen Ou, Shuchen Wang, Wuhua Ma, Dehui Fan

**Affiliations:** ^1^ The Fifth Clinical Medical School, Guangzhou University of Chinese Medicine, Guangzhou, China; ^2^ Department of Oncology Rehabilitation, Jincheng People’s Hospital, Jincheng, China; ^3^ Department of Anesthesiology, The First Affiliated Hospital of Guangzhou University of Chinese Medicine, Guangzhou, China; ^4^ Department of Anesthesiology, Jincheng People’s Hospital, Jincheng, China; ^5^ Department of Rehabilitation, GuangDong Second Traditional Chinese Medicine Hospital, Guangzhou, China

**Keywords:** ceRNA, immune microenvironment, SCNA, genetic variants, methylation

## Abstract

**Background:** Gastric cancer (GC) is the second leading cause of cancer-related mortality and the fifth most common cancer worldwide. However, the underlying mechanisms of competitive endogenous RNAs (ceRNAs) in GC are unclear. This study aimed to construct a ceRNA regulation network in correlation with prognosis and explore a prognostic model associated with GC.

**Methods:** In this study, 1,040 cases of GC were obtained from TCGA and GEO datasets. To identify potential prognostic signature associated with GC, Cox regression analysis and the least absolute shrinkage and selection operator (LASSO) regression were employed. The prognostic value of the signature was validated in the GEO84437 training set, GEO84437 test set, GEO15459 set, and TCGA-STAD. Based on the public databases, TargetScan and starBase, an mRNA-miRNA-lncRNA regulatory network was constructed, and hub genes were identified using the CytoHubba plugin. Furthermore, the clinical outcomes, immune cell infiltration, genetic variants, methylation, and somatic copy number alteration (sCNA) associated with the ceRNA network were derived using bioinformatics methods.

**Results:** A total of 234 prognostic genes were identified. GO and GSEA revealed that the biological pathways and modules related to immune response and fibroblasts were considerably enriched in GC. A nomogram was generated to provide accurate prognostic outcomes and individualized risk estimates, which were validated in the training, test dataset, and two independent validation datasets. Thereafter, an mRNA-miRNA-lncRNA regulatory network containing 4 mRNAs, 22 miRNAs, 201 lncRNAs was constructed. The KCNQ1OT1/hsa-miR-378a-3p/RBMS1 ceRNA network associated with the prognosis was obtained by hub gene analysis and correlation analysis. Importantly, we found that the KCNQ1OT1/miR-378a-3p/RBMS1 axis may play a vital role in the diagnosis and prognosis of GC patients based on Cox regression analyses. Furthermore, our findings demonstrated that mutations and sCNA of the KCNQ1OT1/miR-378a-3p/RBMS1 axis were associated with increased immune infiltration, while the abnormal upregulation of the axis was primarily a result of hypomethylation.

**Conclusion:** Our findings suggest that the KCNQ1OT1/miR-378a-3p/RBMS1 axis may be a potential prognostic biomarker and therapeutic target for GC. Moreover, such findings provide insights into the molecular mechanisms of GC pathogenesis.

## Introduction

Globally, the incidence of gastric cancer (GC) is increasing. In fact, GC has the fifth highest incidence and second highest incidence for cancer-related mortality (8.8% of cancer deaths). Accordingly, GC remains a significant global health problem (McAuliffe et al., 2019). As the disease is largely asymptomatic, the absence of clinical signs delays diagnosis. Three quarters of patients have non-curable advanced disease, leading to poor prognosis (Seidlitz et al., 2019). The primary treatment for GC includes surgery, radiation, chemotherapy, hormone therapy, immuno-therapy, and targeted therapy (Rasool et al., 2013). However, owing to clinical heterogeneity between patients, the treatment and prediction of survival outcomes are challenging as patients with the same diagnosis often have different responses to treatment. Many studies with different biomarkers for predicting early detection, treatment response, and overall survival (OS) have been published (Brahmer et al., 2015; The International CLL-IPI working group, 2016). Accordingly, new therapeutic targets and prognostic biomarkers for this deadly disease are urgently needed.

Integrative bioinformatics analysis has been identified as a useful tool for revealing prognostic biomarkers and selecting an appropriate treatment approach. Large-scale genomic projects, such as The Cancer Genome Atlas (TCGA) and Gene Expression Omnibus (GEO), have provided new tools for identifying gene drivers and therapeutic targets for cancer (Hu et al., 2019; Lang et al., 2020). The widespread availability of high-throughput sequencing plays a critical role in the development of novel diagnostic and therapeutic strategies and the early diagnosis and treatment of diseases (Liu et al., 2017).

Numerous studies have focused on single-gene predictions of disease risk; however, it is uncertain to predict prognosis. Based on recent studies, polygenic risk scores may result in a more reliable and robust predicted response than single-gene prediction (Purcell et al., 2009; Chatterjee et al., 2016; Shi et al., 2016). For example, polygenic risk scores estimate that the absolute risk increases in carriers of BRCA1 and BRCA2, which might influence clinical decision making (Kuchenbaecker et al., 2017). Furthermore, polygenic risk scores have shown promise for the prediction of multiple common diseases, including coronary artery disease, prostate cancer, breast cancer, and type 2 diabetes (Lambert et al., 2019).

Long non-coding RNAs (lncRNAs) are non-protein coding RNAs with a length of at least 200 nucleotides. As active regulators of coding gene expression, lncRNAs are becoming important players in cancer progression and invasion (Schmitt and Chang, 2016; Arnes et al., 2019). MicroRNAs (miRNAs) are small, noncoding RNAs that bind to target mRNAs in their 3’-untranslated region (3’UTR). miRNAs inhibit the translation or promote degradation of mRNA, which reduces protein expression (Ouimet et al., 2016). Accordingly, miRNAs play a critical role in the progression of cancers, including GC (Si et al., 2018). LncRNAs have been shown to act as a “sponge” for miRNAs, a process known as endogenous competing RNA (ceRNA), which reduces the suppressive effect of miRNAs on target mRNAs (Karagkouni et al., 2020). For example, lncRNA-miRNA-mRNA ceRNA networks might participate in the initiation and progression of cancers, and may be a target for early diagnosis, prognosis evaluation, and treatment (Shih et al., 2020; Wang et al., 2020; Zhang and Lou, 2020).

In this study, we constructed a ceRNA regulation network related to prognosis and explored a signature prognostic model in 1,040 patients with GC. First, both differential expression genes and potential prognostic genes were identified from two datasets. Based on LASSO regression, a multigene prognostic signature was developed and validated using three independent datasets. Second, the mRNA-miRNA-lncRNA regulatory network associated with prognosis was identified using public databases. Through hub gene analysis, a key ceRNA network was obtained. Third, the association between the ceRNA network and clinical outcomes was assessed, which revealed that the KCNQ1OT1/RBMS1 axis may be vital for the diagnosis and prognosis of GC patients. Finally, the relationships between the KCNQ1OT1/RBMS1 axis and immune cell infiltration, genetic variants, methylation, and somatic copy number alteration (sCNA) were evaluated to explore the possible biological function of the KCNQ1OT1/RBMS1 axis in GC.

## Materials and Methods

### Gastric Cancer Dataset Sources

**FIGURE 1:**
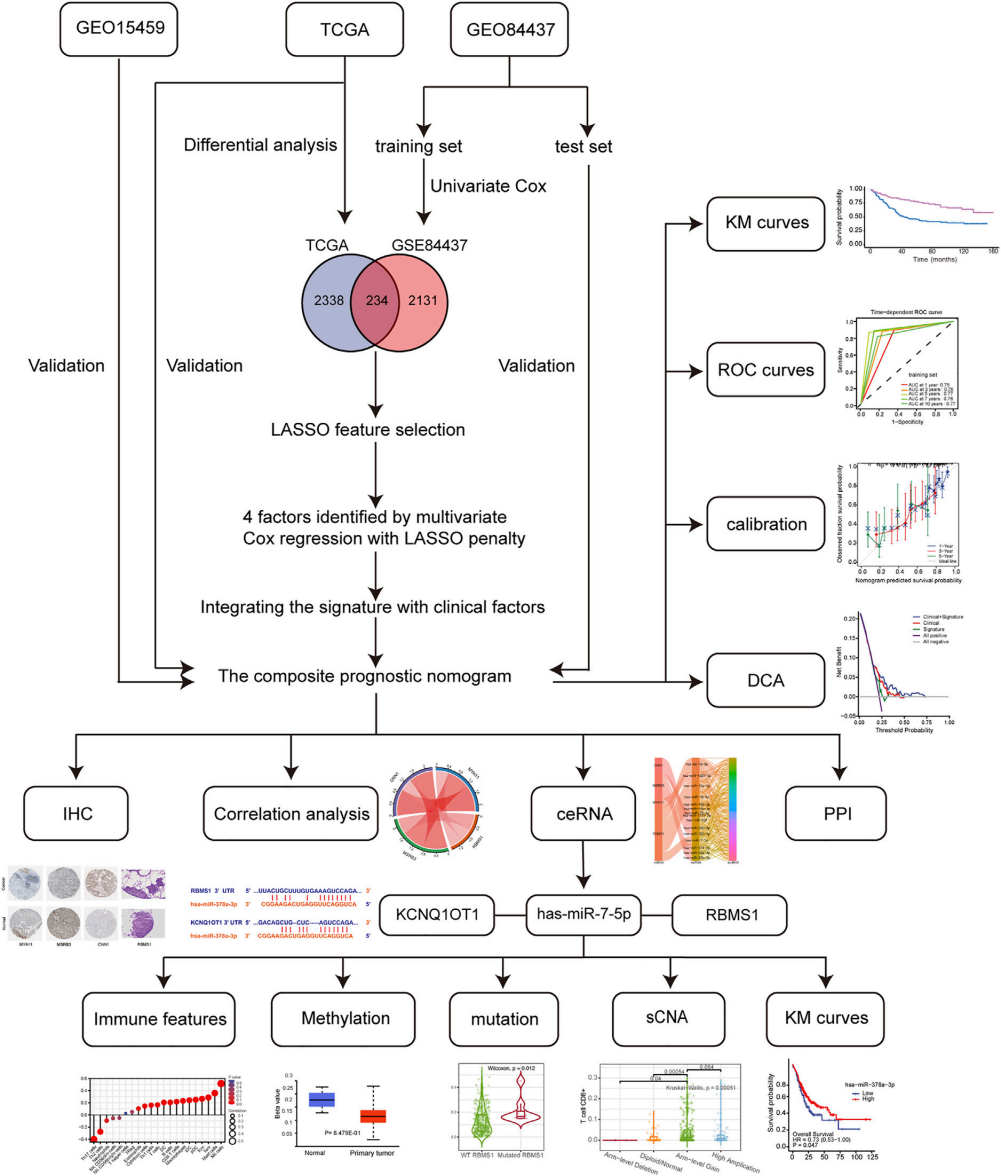
Flowchart of the study design, including data collection and analysis.

The flowchart (Figure 1) shows the most important steps in the analysis process. TCGA and GEO databases were searched systematically to obtain public gene expression data and complete clinical annotations. For further analysis, cohorts that had the following characteristics were excluded: 1) less than 200 patients, 2) lack of raw CEL files, 3) lack of basic clinical information [sex, age, tumor-node-metastasis (TNM) stage), or 4] lack of survival information. Three eligible GC public datasets (GSE84437, GSE15459, and TCGA-STAD) were then analyzed.

GSE84437, measured using the Illumina HumanHT-12 V3.0 expression Beadchip, consisted of 433 GC samples (Yoon et al., 2020). The GSE84437 cohort was treated as a discovery set and randomly (in a 1:1 ratio) classified into two subgroups: the training set and test set. The training set was used to develop a prognostic model, and the test set was used for internal validation of the prognostic model. GSE15459, measured using the Affymetrix Human Genome U133 Plus 2.0 Array, contained 200 GC samples (Subhash et al., 2018). The TCGA-STAD dataset included 375 GC samples and 32 normal samples. The GSE15459 and TCGA-STAD were used as independent validation sets (external validation). The validation sets were used to evaluate the predictive ability of prognostic model.

### Data Preprocessing

The raw CEL files for microarray data were downloaded from the GEO database. The R packages, “affy” and “lumi,” were used to calculate background-adjusted, quantile-normalized, and probe-level data-summarized values for all probe sets (Irizarry et al., 2003; Gautier et al., 2004; Du et al., 2008). The “ComBat” algorithm in the “sva” package was used to correct batch effects from nonbiological technical biases (Leek et al., 2012).

Level 3 RNAseq FPKM files for stomach adenocarcinoma (STAD) cases were downloaded from TCGA (https://portal.gdc.cancer.gov/). The RNAseq data were converted from FPKM to TPM format and then log2 transformed, leading to data that are more comparable to microarrays and enable comparison between samples. Based on the GPL570 platform, gene symbols were annotated to the Affymetrix probe ID from the microarray data. Based on the GPL6947 platform, gene symbols were annotated to the Illumina probe ID from the microarray data. The mean expression value was considered for probes that mapped to a single gene.

### Identification of Prognostic Genes

Genes associated with OS (Cox univariate *p*-value < 0.05) were identified using the “survminer” and “survival” packages (Liu et al., 2018). Differential expression analysis was performed on the TCGA-STAD dataset using the “limma” package (Ritchie et al., 2015). To evaluate as many genes as possible and enhance statistical power, differentially expressed genes (DEGs) were selected based on an adjusted *p* value < 0.05. Prognostic genes were identified by the intersection of the two sets of genes after the above screening process.

### Enrichment Analysis of Functions and Pathways

Gene Ontology (GO) and Kyoto Encyclopedia of Genes and Genomes (KEGG) pathway enrichment analyses were performed using the “clusterProfiler” package (Yu et al., 2012). Gene Set Enrichment Analysis (GSEA) was also performed to identify pathways enriched among DEGs. Using the “clusterProfiler” and “org.Hs.eg.db” packages, we determined the molecular pathways that differed significantly between GC tissues and normal tissues. We analyzed the Reactome pathways with GSEA and used the “clusterProfiler” package to identify each functional cluster. The reference gene set was C2. all.v6.2. symbols.gmt (Newman and Weiner, 2005). *p*-value < 0.01 and a false discovery rate <0.1 were set as the cut-off criteria.

### Construction and Verification of the Prognostic Model

To identify the best prognostic genes to maximize predictive ability, Lasso regression was performed using the “glmnet” package with default settings to screen out final prognostic genes (Lossos et al., 2004). A Cox regression multivariable analysis was conducted based on final prognostic genes. Prognostic signature was generated using the regression coefficient of the model. Gene expression levels were weighted by their respective coefficients of Cox regression to calculate the signature’s risk score. Based on the following formula, the risk score was calculated for the signature:

$$Risk\;Score=\overset{n}{\underset{i=1}{\sum }}Coef_{i}\times Exp_{i}$$

Where n , $Coef_{i}$ , and $Exp_{i}$ represent the number of selected gene, coefficient, and the expression value of each selected gene, respectively. A combined risk score was derived from this multivariable model, and the median value of the risk score was used to divide patients into high- and low-risk categories. The Log-rank test and Kaplan-Meier method were used to analyze survival rates.

To establish the prognostic model of GC, we included age, gender, cancer stage, and prognostic-related risk score. For further validation, the corresponding regression coefficients were estimated from the training set and validated using the test set and the other validation sets (TCGA and GEO15459). To evaluate the prognostic performance of the model, the “survivalROC” package was used to construct time-dependent receiver operating characteristic (ROC) curves (Li et al., 2018). Finally, to visualize and apply the model, we constructed a nomogram that included age, gender, T stage, N stage, and the multigene signature. Calibration curves and decision curve analysis (DCA) were used to evaluate the clinical application prospects of nomograms (Vickers and Elkin, 2006).

### Correlation Analysis Between Prognostic Genes and PPI Network

Physical and functional interactions of proteins can be predicted based on the protein-protein interaction (PPI) information in the STRING database (https://cn.string-db.org/) (Szklarczyk et al., 2021). We selected the protein nodes with the strongest connectivity in Cytoscape software (version 3.9.1, https://cytoscape.org/) for visualization of molecular interaction networks (Shannon et al., 2003). The Spearman’s correlation test and “ggplot2” package were used to analyze the correlation between prognostic genes. Furthermore, pairwise correlations between prognostic genes were visualized as a chord diagram using the “circlize” package.

### Immunohistochemical Analysis

The Human Protein Atlas (HPA) database (version: 21.0, http://www.proteinatlas.org/) is the most comprehensive database for assessing protein distribution in human tissues (Uhlén et al., 2015). Protein expression data of prognostic genes were gathered from the HPA database. The protein expression of genes was analyzed using immunohistochemical staining images in normal and GC tissues.

### Construction of the mRNA-miRNA-lncRNA Network

To develop the mRNA-miRNA-lncRNA regulatory network, the starBase version 2.0 database (http://starbase.sysu.edu.cn) (Li et al., 2014) and TargetScan (version 7.2, http://www.targetscan.org/) (Agarwal et al., 2015) were used to identify potential miRNAs interacting with prognostic genes and their downstream target lncRNAs. The criteria used to select these miRNAs were results with a high stringency of CLIP data >2 and results with a used predicting program >3. The generated networks were visualized using Cytoscape (Shannon et al., 2003). The Sankey diagram was visualized to further reveal the relationship between the mRNA-miRNA-lncRNA regulatory network using the “ggalluvial” package. A hub regulatory network for the merged network was obtained through CytoHubba plug-in (Chin et al., 2014). To perform a more comprehensive analysis of the effects on the ceRNA network, differential expression and survival analysis for hub genes were carried out using the “ggplot2” and “survminer” packages. Further, miRNA-target co-expression analysis was performed to explore the correlation between all hub genes using the “ggplot2” package. Finally, mRNA and lncRNA target sites were predicted for pairing with miRNA using the TargetScan and starBase databases.

### Immune Infiltration, Genetic Variants, Methylation, and sCNA

Tumor infiltrating lymphocytes (TILs) have been identified as a reliable predictor of OS (Azimi et al., 2012). We examined the relationships between the ceRNA network and infiltrating immune cells in GC. We also obtained marker genes for the immune cell types involved in ssGSEA from the studies of Bindea et al. and Senbabaoglu et al. (Bindea et al., 2013). Using the “gsva” package, the infiltration levels of different types of immune cells were quantified (Hänzelmann et al., 2013). Total T-cell Infiltration Score and Immune Infiltration Score were calculated using the ssGSEA scores for each type of immune cell. The relationships between the prognostic genes and infiltrating immune cells in GC were analyzed using TIMER 2.0 (Li et al., 2020) and UCLCAN (Chandrashekar et al., 2017). Moreover, the correlation between immune infiltration and mutation profile, methylation, and sCNA data, which provided insights into the potential mechanisms of prognostic genes in GC, was determined.

### Statistical Analysis

Statistical analysis was performed using the R software (version 3.6.3). Continuous data were analyzed using Mann-Whitney tests and categorical data were analyzed using Fisher’s exact tests. Pearson correlation coefficient was used to estimate the correlation between continuous variables. The Kaplan-Meier method was used for survival analysis. The Log-rank test was used to determine the significance of differences. All statistical analyses were considered significant if the *p*-values were less than 0.05.

## Results

### Identification of Prognostic Genes

**FIGURE 2:**
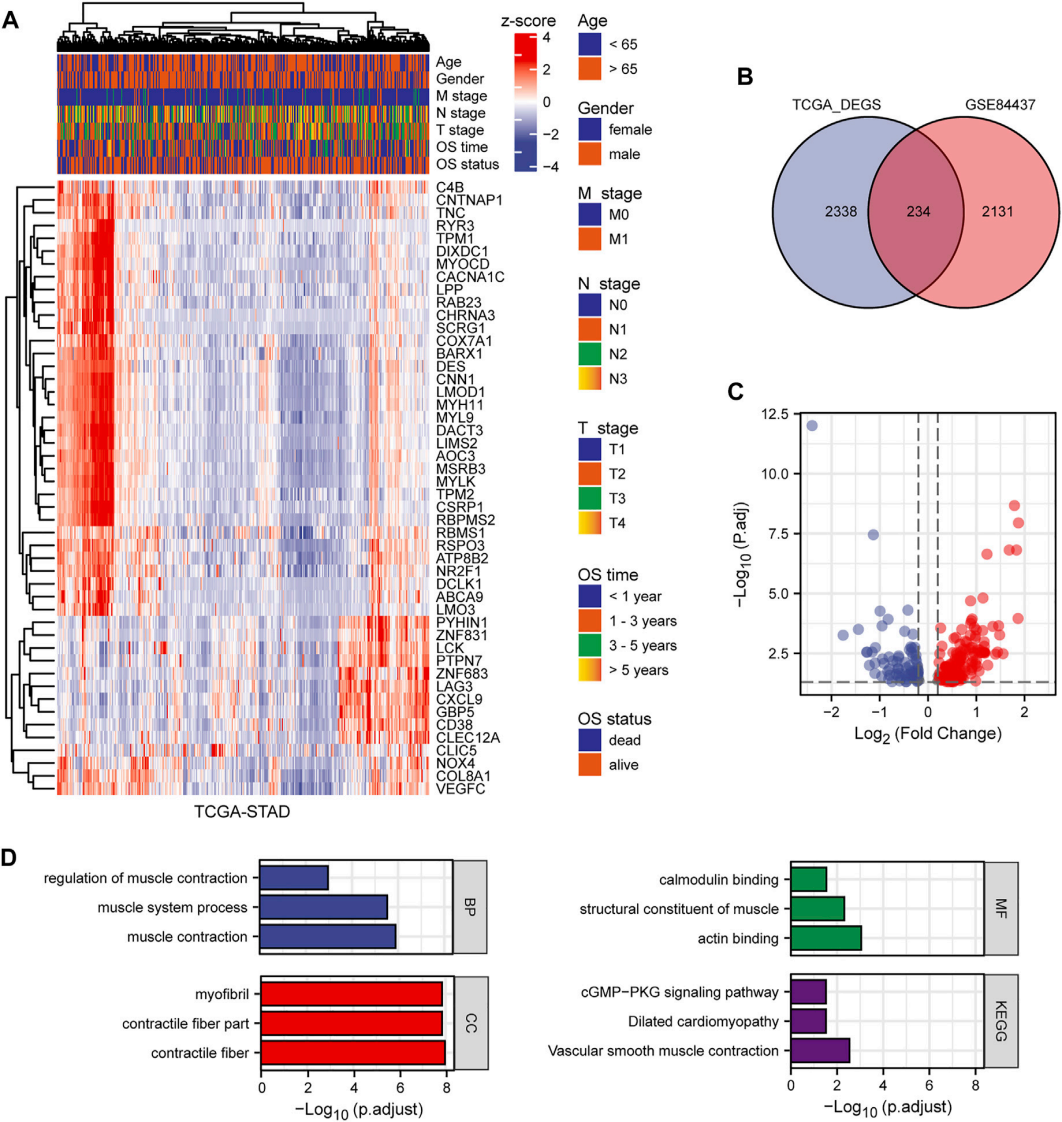
The results of differential expression analysis. **(A)** Heat map of the top 50 differentially expressed genes. **(B)** Venn diagram showing the intersection of TCGA-STAD and GSE84437. **(C)** Volcano plot showing the results of differentially expressed genes. **(D)** GO and KEGG enrichment of differentially expressed genes.

This study included 1,040 patients with GC from three independent datasets (GSE84437, GSE15459, and TCGA-STAD). By standardizing and removing batch effects from the microarray results, a total of 2,365 prognostic genes in GSE84437 and 2,572 differentially expressed genes in TCGA-STAD datasets were identified. The top 50 differentially expressed genes were extracted and a heat map was generated according to the log fold change (Figure 2A). The Venn diagram in Figure 2B was constructed to show the intersection of the two datasets. Volcano plots were used to visually represent the differential analysis results (Figure 2C).

### Functional Analysis of Prognostic Genes

We analyzed the GO and KEGG functional terms enriched among DEGs using the “clusterProfiler” package. The DEGs were enriched in Z disc, contractile fiber, and contractile fiber part terms for cellular component (CC). For biological process (BP), the DEGs were enriched in actomyosin structure organization, muscle contraction, and muscle system process terms. For molecular function (MF), the DEGs were enriched in FAD binding, flavin adenine dinucleotide binding, and actin binding terms. Three KEGG pathways were differentially enriched, including biosynthesis of unsaturated fatty acids, tight junction, and vascular smooth muscle contraction (Figure 2D). Using the significantly enriched GO and KEGG terms, potential biomarkers for GC may be identified.

### GSEA of Prognostic Genes

**FIGURE 3:**
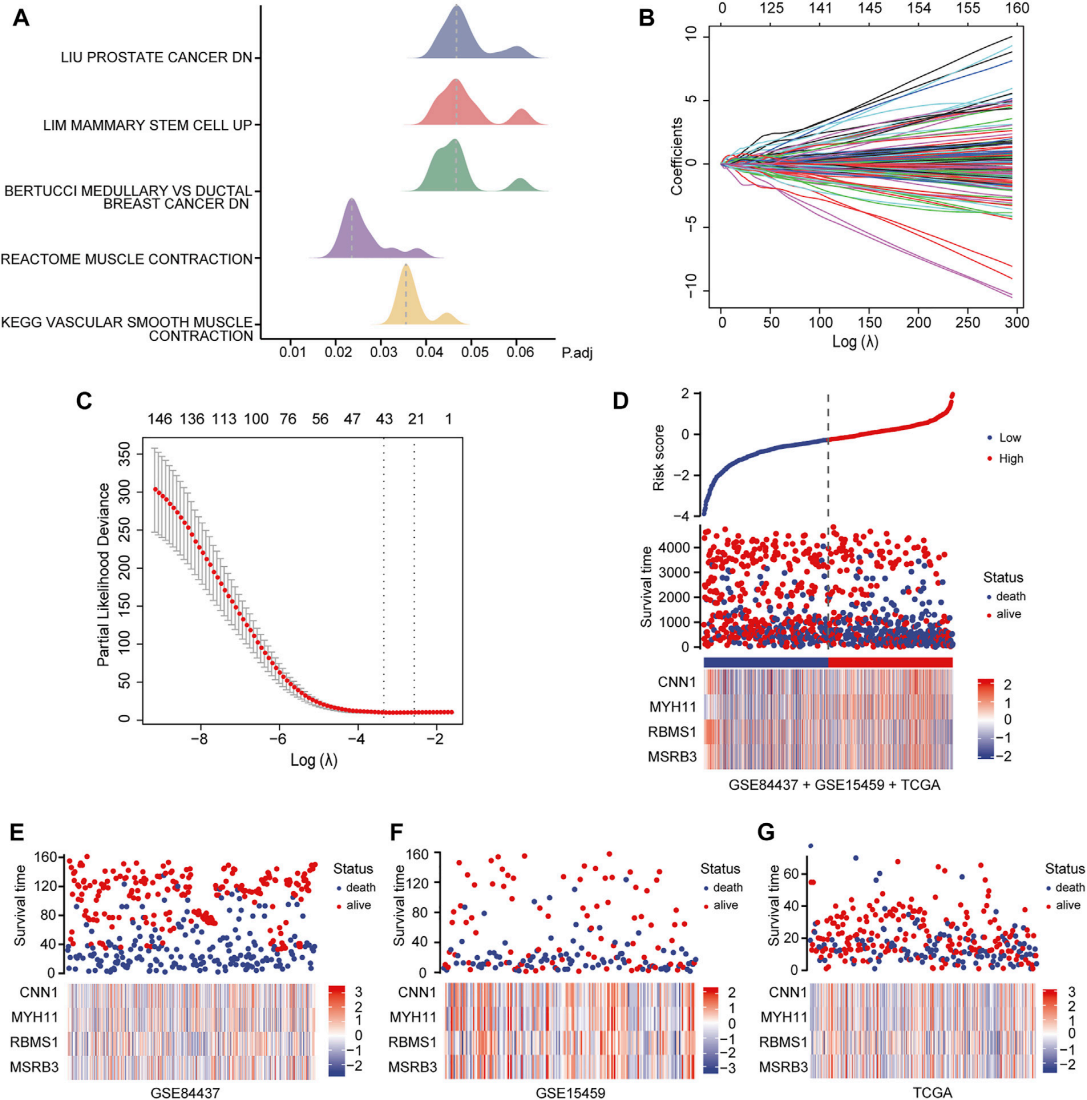
Identification and analysis of prognostic genes. **(A)** GSEA of GC patients and non-GC patients. **(B, C)** LASSO regression analysis of 234 candidate genes. **(D)** Heat map showing the risk scores of the high and low-risk groups for the four prognostic genes in all three datasets. **(E)** Risk scores for the high and low-risk groups for four prognostic genes in the GSE84437 dataset. **(F)** Risk scores for the high and low-risk groups for four prognostic genes in the GSE15459 dataset. **(G)** Risk scores for the high and low-risk groups for four prognostic genes in the TCGA dataset.

We identified the differentially expressed signaling pathways in GC patients and those without GC using GSEA. Patients with GC showed significant changes in molecular pathways compared with those without GC. These analyses revealed that pathways related to GC primarily included muscle contraction pathways, prostate cancer, mammary stem cells, medullary vs ductal breast cancer, and vascular smooth muscle contraction (Figure 3A).

### Patient Characteristics

The training set consisted of 213 GC patients, including 68 (31.92%) females and 145 (68.08%) males; the median age was 59.63 years (Supplementary Material S1). In the test set, 213 patients with GC were included; 67 (31.46%) were females, and 146 (68.54%) were males; the median age was 60.28 years (Supplementary Material S1). Among the 182 GC patients in the independent validation set, GSE15459, 66 (36.26%) were females, and 116 (63.74%) were males; the median age was 66.45 years (Supplementary Material S2). The independent validation set, TCGA-STAD, included 407 patients, of which 144 (35.38%) were females, and 263 (64.62%) were males; the median age of these patients was 66.04 years (Supplementary Material S3).

### Identification of Prognostic Genes

To identify the best prognostic genes to maximize predictive ability, we employed the Lasso Cox regression algorithm for the 234 candidate genes using the “glmnet” package with default settings (Figures 3B,C). According to the results of the Lasso-selected features, Calponin 1 (*CNN1*), Myosin Heavy Chain 11 (*MYH11*), RNA Binding Motif Single Stranded Interacting Protein 1 (*RBMS1*), and Methionine Sulfoxide Reductase B3 (*MSRB3*) were identified and added to the multigene signature that predicts the survival of GC patients. The following formula was used to estimate the risk scores for each patient in all three datasets:

Risk score=0.344×CNN1-0.086×MYH11-0.068×RBMS1-0.254×MSRB3

**FIGURE 4:**
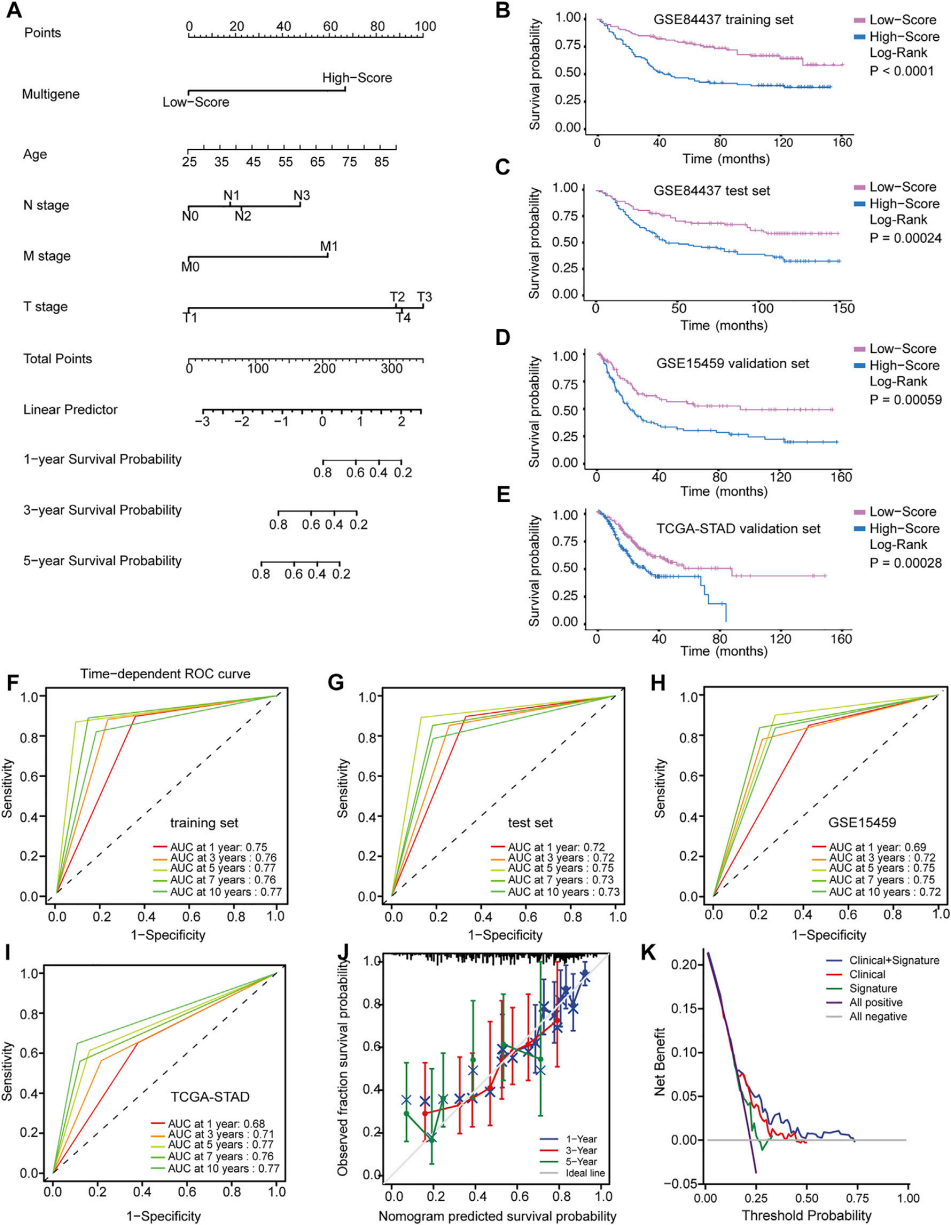
Identification and application of the multigene signature. **(A)** Nomogram predicting the probability of survival for GC patients at 1, 3 and 5 years **(B–E)** Kaplan-Meier curves of the training and validation datasets on overall survival. **(F–I)** Time-dependent ROC curves of nomogram in the training and validation datasets. **(J)** Calibration curve of predicted results compared to actual observation results. **(K)** DCA demonstrating the net benefit of nomogram, clinical features model, and multigene signature.

Patients in all three datasets (GSE84437, GSE15459, and TCGA-STAD) were then grouped into high- and low-risk groups using the median risk scores as the cutoff. The risk scores for the four genes were visualized as heat maps in the high-risk and low-risk groups (Figures 3D–G). A higher mortality rate and worse prognosis were identified for the high-risk-score groups than for the low-risk-score groups. Kaplan-Meier survival curves indicated that patients with low-risk scores had a significantly longer OS than patients with high-risk scores (GSE84437 training set: Log-rank *p* < 0.0001; GSE84437 validation set: Log-rank *p* = 0.00024; GSE15459 validation set: Log-rank *p* = 0.00059; TCGA-STAD validation set: Log-rank *p* = 0.00028) (Figures 4B–E).

### Clinical Application of the Multigene Signature

Although these prognostic genes are independently prognostic of OS, clinical characteristics, such as age and TNM stage, may also play an independent prognostic role in multivariable models. In the training and validation datasets, Cox regression analysis was used to conduct univariate and multivariate survival analyses. All significant variables in the univariate Cox regression (*p* < 0.05) were further analyzed in a multivariate Cox regression to identify independent prognostic factors. Finally, the multigene signature was integrated with these clinical variables to construct a nomogram that predicted the 1-, 3-, and 5-years survival probability of GC patients (Figure 4A).

Based on the ROC curve and AUC values, the accuracy of the nomogram model’s predictions was determined. The time-dependent ROC curve in Figures 4F–I suggested that across all datasets, the nomogram was a good predictor of OS for GC patients. Predictions and actual observations agreed well with the calibration curve (Figure 4J). Moreover, DCA was performed to compare the net benefit of the nomogram model with that of the clinical features model and multigene signature model (Figure 4K). Compared to the other two models, the nomogram model had a greater clinical net benefit. These findings were verified via internal validation and two independent external validation datasets, suggesting the reliability and efficiency of our nomogram as a prognostic model.

### Correlation Analysis Between Four Prognostic Genes and PPI Network

**FIGURE 5:**
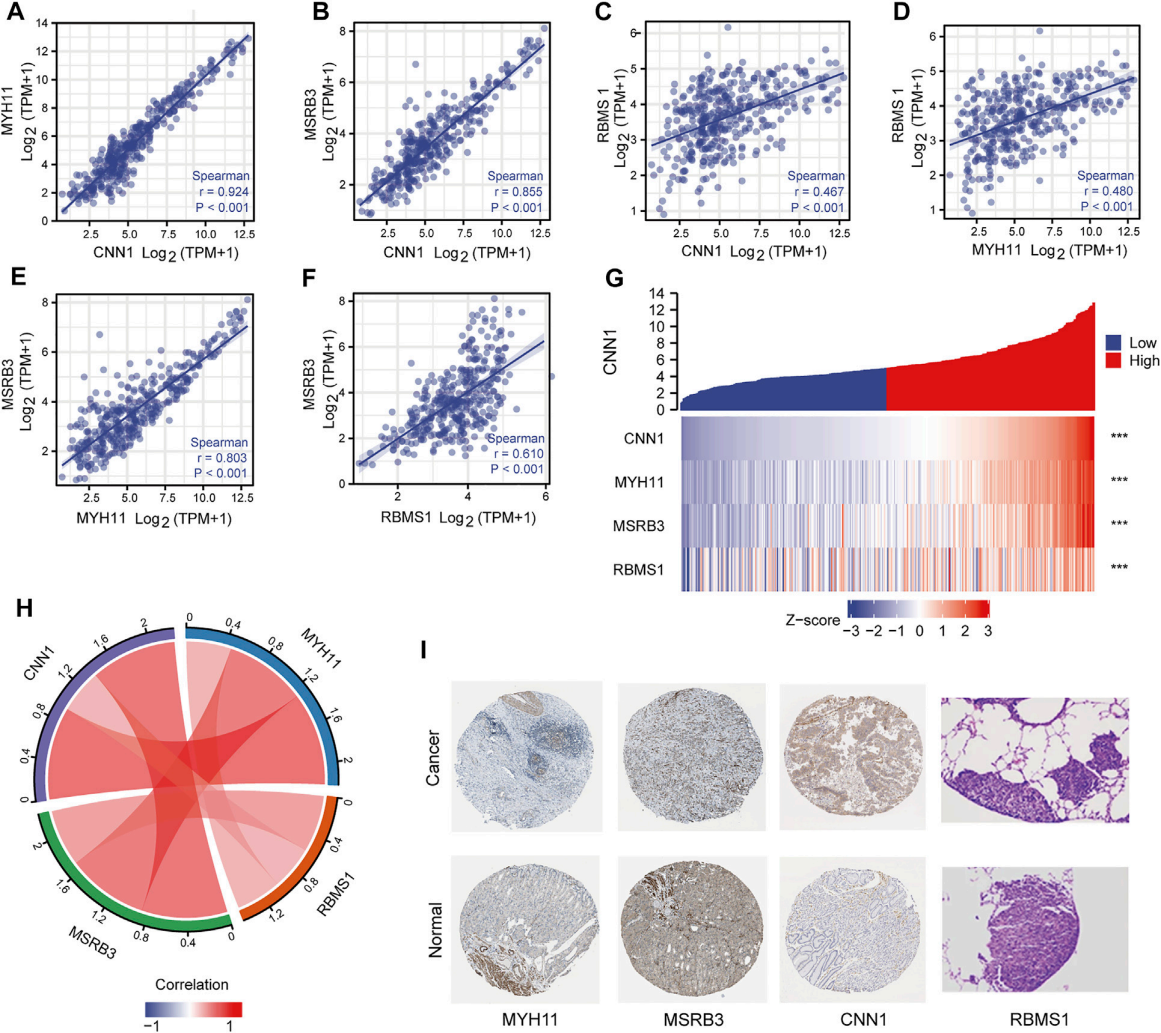
Correlation analysis between four prognostic genes. **(A–F)** Scatter plots showing the correlation between four prognostic genes. **(G,H)** Chord diagram and heatmap visualizing the correlation between four prognostic genes. **(I)** Immunohistochemical analysis of four prognostic genes.

As shown in Figure 5, a strong correlation among CNN1, MYH11, and MSRB3, was observed, with r > 0.8 (Figures 5A–D). The moderate correlation between RBMS1 and the other three genes was observed with 0.5 < r < 0.8 (Figures 5E,F). Pairwise correlations between four prognostic genes were visualized as a chord diagram and a heatmap (Figures 5G,H). Further, a PPI network was constructed by analyzing the interactions between four prognostic genes using the STRING database. Visualization of the results was performed with Cytoscape (Supplementary Material S4).

### Immunohistochemical Analysis

Immunohistochemical analysis of the HPA database indicated that the protein products of risk-associated genes were expressed at higher levels in GC samples than in normal samples (Figure 5I). Immunohistochemical images of RBMS1 were obtained from a recent study (Liu et al., 2022).

### Construction of the mRNA-miRNA-lncRNA Regulatory Network

**FIGURE 6:**
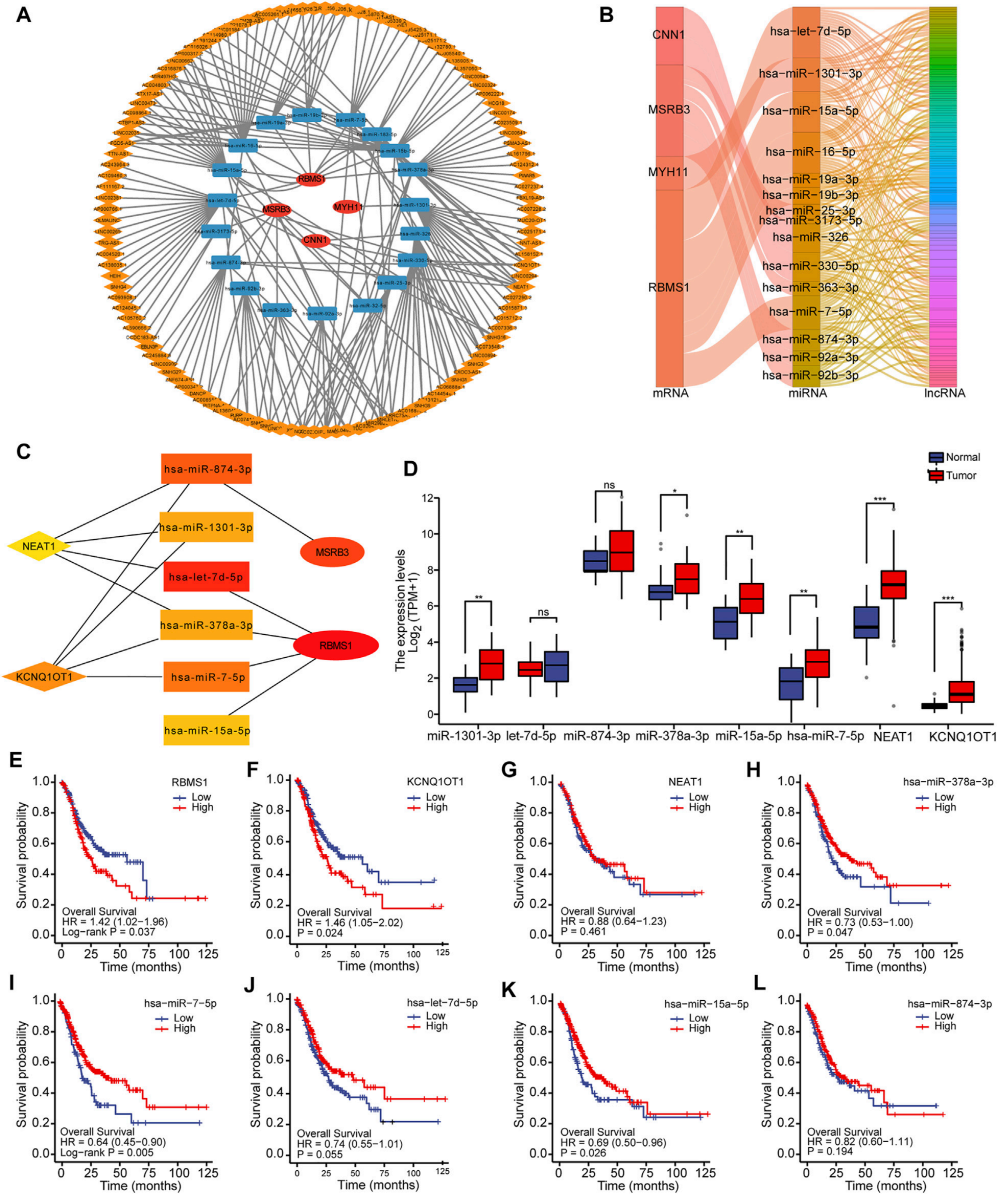
Construction of the mRNA-miRNA-lncRNA regulatory network. **(A)** Construction of the ceRNA network associated with GC. **(B)** Sankey diagram for ceRNA network visualization. **(C)** Identification of the hub network for further analysis. **(D)** Box plots showing the expression levels of miRNAs and lncRNAs in hub genes. **(E–L)** Kaplan-Meier curves showing the overall survival based on the top hub genes.

Using the starBase database (version 2.0), the network of mRNA-miRNA-lncRNAs, as well as the downstream target lncRNAs, was established based on potential miRNA interactions with four prognostic genes (*CNN1*, *MYH11*, *MSRB3,* and *RBMS1*). A total of 4 mRNAs, 22 miRNAs, and 201 lncRNAs were included to generate the ceRNA network associated with GC (Figure 6A). Sankey diagram was employed to further visualize the relationship between the mRNA-miRNA-lncRNA regulatory network using the “ggalluvial” package (Figure 6B). The hub network was obtained using the CytoHubba plug-in (Figure 6C).

To more comprehensively determine the effects on the ceRNA network, differential expression and survival analysis were performed using the 10 hub genes. The expression level of miRNAs and lncRNAs for the top 10 hub genes was depicted using box plots (Figure 6D). Based on Kaplan-Meier analysis, the OS rate was significantly lower in GC patients with high-risk scores for mRNA and lncRNA, while the OS rate for miRNA was significantly higher, as shown in Figures 6E–L. One lncRNA (KCNQ1OT1), two miRNAs (miR-378a-3p and miR-7-5p), and one mRNA (RBMS1) were identified to be associated with prognosis.

**FIGURE 7:**
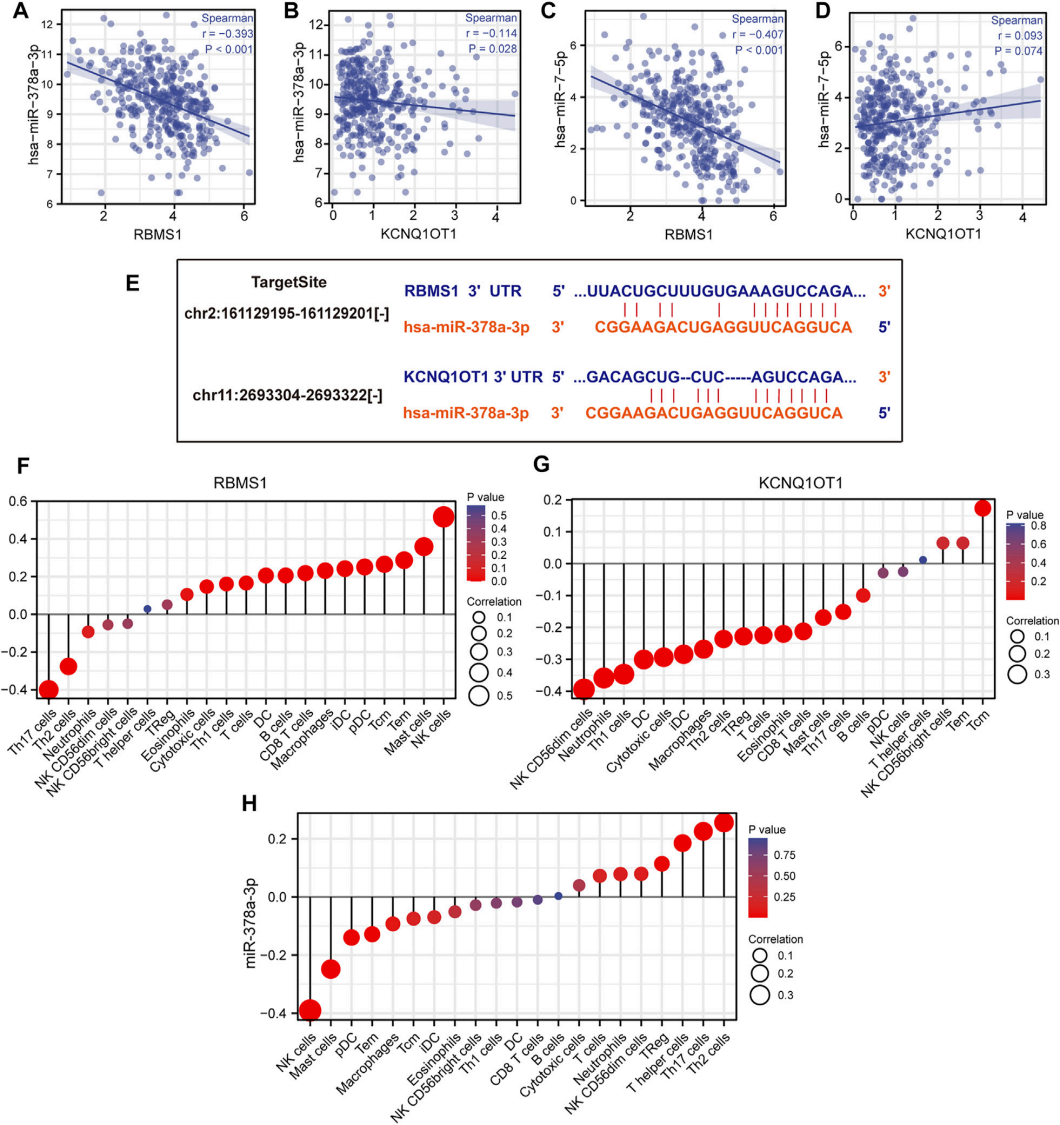
Identification of the KCNQ1OT1/miR-378a-3p/RBMS1 axis. **(A–D)** Scatter plots showing miRNA-target co-expression of hub genes associated with prognosis. **(E)** Binding site prediction for the KCNQ1OT1/miR-378a-3p/RBMS1 axis. **(F–H)** Correlation analysis of the KCNQ1OT1/miR-378a-3p/RBMS1 axis with infiltrating immune cells.

The results of miRNA-target co-expression suggested that weak-moderate negative correlations existed between miRNA and its target in four hub genes based on Spearman correlation, except for has-miR-7-5p (*p* > 0.05) (Figures 7A–D). These results suggest that KCNQ1OT1 might function as a ceRNA regulating the expression of RBMS1 by sponging miR-378a-3p. Finally, the KCNQ1OT1/miR-378a-3p/RBMS1 axis was identified and the KCNQ1OT1 and RBMS1 target sites were predicted to pair with miR-378a-3p using the TargetScan and starBase databases (Figure 7E).

### Clinical Relevance of the KCNQ1OT1/miR-378a-3p/RBMS1 Axis in GC

We proceeded to determine the clinical significance of RBMS1, KCNQ1OT1, and miR-378a-3p at GC diagnosis and whether their expression levels were influenced by clinical characteristics. The expression of RBMS1 was found to be positively correlated with pathologic stage, N stage, and T stage, while KCNQ1OT1 and miR-378a-3p had weak correlations with age and TNM stage (Supplementary Material S5). Univariate and multivariate Cox regression analyses were also performed to determine the prognostic significance of RBMS1, KCNQ1OT1, miR-378a-3p, and clinical features.

Importantly, a significant association was found between RBMS1^high^ expression, age, M stage, and T stage in TCGA-STAD cohorts (Table 1). However, the expression of KCNQ1OT1 and miR-378a-3p was not associated with a poor prognosis (Table 2, 3). Overall, RBMS1 expression level may serve as an independent prognostic factor for OS in GC patients.

**TABLE 1:** Univariate and multivariate Cox analysis of clinical variables and RBMS1.

Characteristics	Total (N)	Univariate Analysis		Multivariate Analysis	
		Hazard ratio (95% CI)	*p* Value	Hazard ratio (95% CI)	*p* Value
RBMS1	370				
Low	182	References			
High	188	1.418 (1.017–1.976)	**0.039**	1.430 (1.008–2.030)	**0.045**
Age	367				
≤65	163	References			
>65	204	1.620 (1.154–2.276)	**0.005**	1.973 (1.368–2.847)	**<0.001**
Gender	370				
Female	133	References			
Male	237	1.267 (0.891–1.804)	0.188		
M stage	352				
M0	327	References			
M1	25	2.254 (1.295–3.924)	**0.004**	2.857 (1.569–5.203)	**<0.001**
T stage	362				
T1	18	References			
T2	78	6.725 (0.913–49.524)	0.061	5.466 (0.736–40.608)	0.097
T3	167	9.548 (1.326–68.748)	**0.025**	7.411 (1.023–53.676)	**0.047**
T4	99	9.634 (1.323–70.151)	**0.025**	6.718 (0.912–49.476)	0.062

The bold value indicates a statistically significant outcome.

**TABLE 2:** Univariate and multivariate Cox analysis of clinical variables and KCNQ1OT1.

Characteristics	Total (N)	Univariate Analysis		Multivariate Analysis	
		Hazard ratio (95% CI)	*p* Value	Hazard ratio (95% CI)	*p* Value
KCNQ1OT1	370				
Low	184	References			
High	186	1.318 (0.947–1.834)	0.101		
Age	367				
≤65	163	References			
>65	204	1.620 (1.154–2.276)	**0.005**	1.865 (1.297–2.682)	**<0.001**
Gender	370				
Female	133	References			
Male	237	1.267 (0.891–1.804)	0.188		
M stage	352				
M0	327	References			
M1	25	2.254 (1.295–3.924)	**0.004**	2.612 (1.441–4.735)	**0.002**
T stage	362				
T1	18	References			
T2	78	6.725 (0.913–49.524)	0.061	6.340 (0.858–46.829)	0.070
T3	167	9.548 (1.326–68.748)	**0.025**	8.514 (1.182–61.341)	**0.034**
T4	99	9.634 (1.323–70.151)	**0.025**	7.704 (1.051–56.446)	**0.044**

The bold value indicates a statistically significant outcome.

**TABLE 3:** Univariate and multivariate Cox analysis of clinical variables and miR-378a-3p.

Characteristics	Total (N)	Univariate Analysis		Multivariate Analysis	
		Hazard ratio (95% CI)	*p* Value	Hazard ratio (95% CI)	*p* Value
hsa-miR-378a-3p	440	0.869 (0.751–1.005)	0.059	0.887 (0.755–1.042)	0.144
Age	437				
≤65	198	References			
>65	239	1.472 (1.080–2.005)	**0.014**	1.736 (1.249–2.413)	**0.001**
Gender	440				
Female	155	References			
Male	285	1.026 (0.747–1.409)	0.875		
M stage	420				
M0	390	References			
M1	30	2.571 (1.553–4.258)	**<0.001**	2.862 (1.676–4.886)	**<0.001**
T stage	430				
T1	22	References			
T2	90	7.430 (1.012–54.570)	**0.049**	6.188 (0.831–46.055)	0.075
T3	197	11.221 (1.562–80.606)	**0.016**	9.090 (1.256–65.794)	**0.029**
T4	121	11.753 (1.623–85.129)	**0.015**	9.052 (1.237–66.258)	**0.030**

The bold value indicates a statistically significant outcome.

### Immune Infiltration, Genetic Variants, Methylation, and sCNA

The relationships between the KCNQ1OT1/miR-378a-3p/RBMS1 axis and infiltrating immune cells in GC were evaluated. The lollipop plot showed that *RBMS1* was positively associated with NK cells, Mast cells, Tem, Macrophages, CD8 T cells, and B cell-related immune responses (Figure 7F). The association between miR-378a-3p, KCNQ1OT1, and the immune infiltrate in GC was also assessed. Interestingly, miR-378a-3p showed the opposite expression of the immune cell infiltration to that in *RBMS1* (Figures 7G,H).

**FIGURE 8:**
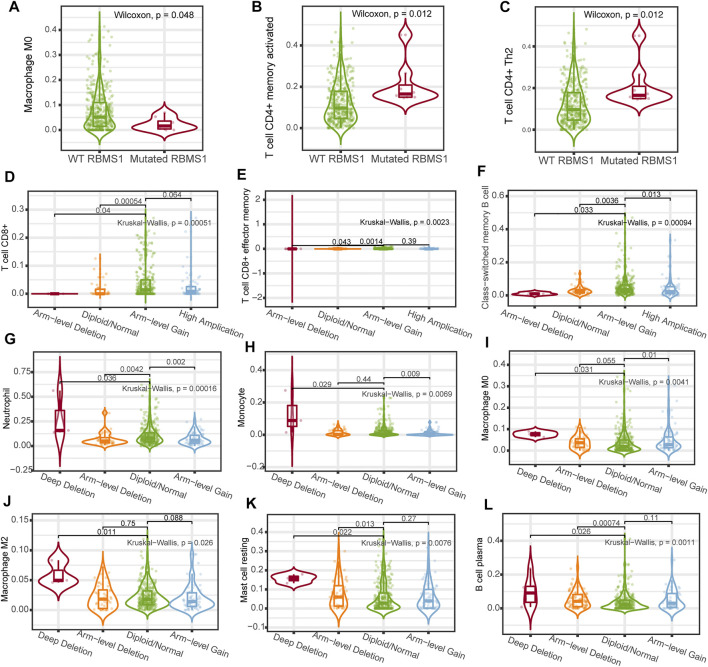
Association between immune cell infiltration and genomic data of the KCNQ1OT1/RBMS1 axis. **(A–C)** Association between immune cell infiltration and RBMS1 mutation. **(D–K)** Relationship between immune cell infiltration and sCNA of RBMS1. **(L)** Relationship between immune cell infiltration and sCNA of KCNQ1OT1.

To better understand the possible mechanisms of the KCNQ1OT1/RBMS1 axis in GC, we analyzed the relationship between immune infiltration and genomic data, such as mutation profile, methylations, and sCNA. The relationships between the KCNQ1OT1/RBMS1 axis and infiltrating immune cells in GC were evaluated using TIMER 2.0 (Li et al., 2020) and UCLCAN (Chandrashekar et al., 2017). Figure 8 shows the results of the analysis. These findings indicate that mutations and sCNA of KCNQ1OT1 and RBMS1 are associated with increased immune infiltration.

**FIGURE 9:**
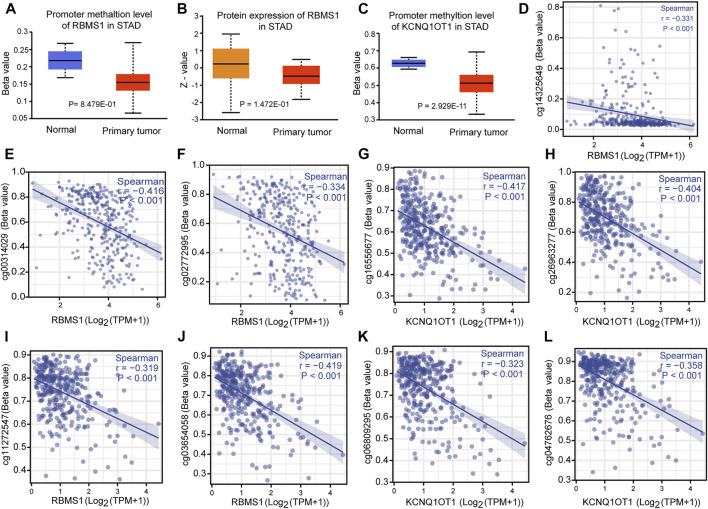
Correlation between methylation and the KCNQ1OT1/RBMS1 axis. **(A,B)** Methylation levels and protein expression of RBMS1 in GC and normal tissues. **(C)** Methylation levels of KCNQ1OT1 in GC and normal tissues. **(D–L)** Correlation between methylation sites and the KCNQ1OT1/RBMS1 axis.

The correlation between genes and their methylation status was determined using different methods. First, UALCAN analysis showed that RBMS1 and KCNQ1OT1 had a tendency for higher methylation levels in normal tissues than in GC tissues (*p* = 0.848 and *p* < 0.0001, Figures 9A–C). Second, the results were virtually identical when the analysis was repeated with DiseaseMeth version 2.0. KCNQ1OT1 and RBMS1 expression levels were also found to be negatively associated with their methylation sites (Figures 9D–L). Third, the heatmaps showed differential methylation regions related to RBMS1 (Supplementary Material S6).

## Discussion

Despite advances in multimodality therapy, GC remains one of the leading causes of cancer deaths worldwide due to its aggressive nature and poor prognosis. Elucidating the molecular mechanisms and processes involved in GC and identifying potential biomarkers may provide clues to new therapeutic targets and influence future therapeutic decisions.

In this study, we revealed the importance of the KCNQ1OT1/miR-378a-3p/RBMS1 axis as a ceRNA regulation network for GC prognosis by analyzing high-throughput sequencing datasets and exploring a multigene prognostic model for tumor-related mortality estimation in GC patients. Most importantly, our exploratory analysis revealed a potential molecular mechanism by which RBMS1 causes poor prognosis in GC, suggesting that RBMS1 may serve as a potential therapeutic target for GC.

The ceRNA regulatory network is reported to play a crucial role in the development and occurrence of many types of cancer. However, only a few studies have focused on a comprehensive ceRNA regulatory network for predicting the prognosis of GC patients. Therefore, we attempted to comprehensively establish a machine-learning model and a ceRNA network associated with prognosis using multiple GEO and TCGA datasets. A total of 234 prognostic genes with differential expression were identified. Further, GO and GSEA revealed that these enriched modules and pathways are closely associated with fibroblasts and immunological responses in GC. Thereafter, a multigene prognostic signature was constructed using LASSO regression. With the multigene signature, GC patients can be classified into high- and low-risk groups. The multigene signature and clinical features were used to generate the nomogram, which was validated using the training and test sets, GEO15459 datasets, and TCGA-STAD. The nomogram provided accurate prognostic outcomes and individualized risk estimates for GC patients.

An mRNA-miRNA-lncRNA regulatory network containing 4 mRNAs, 22 miRNAs, and 201 lncRNAs was obtained using the public databases, TargetScan and starBase. The key ceRNA network, including 2 lncRNAs, 6 miRNAs, and 2 mRNAs, was also obtained using hub gene analysis. Expression analysis and survival analysis were performed using the key ceRNA network. Finally, the clinical outcomes, PPI network, immune cell infiltration, genetic variants, methylation, and sCNA associated with the prognostic sign were identified. Overall, the KCNQ1OT1/hsa-miR-378a-3p/RBMS1 axis was found to be associated with GC prognosis.

Some studies have experimentally studied KCNQ1OT1, miR-378a-3p, and RBMS1 *in vivo* and *in vitro* and verified their expression levels in GC. Zhong et al. (2021) showed that KCNQ1OT1 promotes GC progression via miR-145-5p/ARF6 axis. Li et al. (2022) found that KCNQ1OT1/miR-556-3p/CLIC1 axis may promote GC growth and metastasis. Liu et al. (2022) found that ACTA2-AS1 (lncRNA) suppressed the malignant phenotype of GC cells by targeting the miR-378a-3p/PLCXD2 axis as ceRNA. Liu et al. (2022) revealed the potential molecular mechanism of RBMS1 to promote GC metastasis, suggesting that RBMS1 may be a potential therapeutic target. In addition, Durr et al. (2022) suggested that KCNQ1OT1 and miR-378a may regulate mt-ATP6 content through the ceRNA axis, which may provide a pathway for the treatment of type 2 diabetic heart. These studies validated the expression of KCNQ1OT1/miR-378a-3p/RBMS1 axis in gastric cancer and are consistent with our results in the present study. However, there are still no studies on how the KCNQ1OT1, miR-378a-3p, and RBMS1 axis play a role in the development and progression of gastric cancer.

An lncRNA that acts as an miRNA “sponge” is known as a ceRNA (endogenous competing RNA), which reduces the suppressive effect of miRNAs on target-mRNAs (Karagkouni et al., 2020). Similarly, the results of our analysis suggest that the lncRNA, KCNQ1OT1, acts as a competitive endogenous RNA that competitively binds tumor-suppressive miR-378a-3p, resulting in increased expression of RBMS1 within tumors via the KCNQ1OT1/miR-378a-3p/RBMS1 axis.

Early studies revealed that immune infiltration within the tumor is typically associated with prognosis and response to immunotherapy in many cancers (Fridman et al., 2011; Keenan et al., 2019). However, to date, no studies have evaluated the role of RBMS1 regarding immune infiltrates in the development of GC. In this study, several databases and R packages were used to carry out a comprehensive analysis of the relationship between immune infiltration and GC prognosis. RBMS1 was found to be positively associated with NK cells, Mast cells, Central Memory T cells, and Macrophage-related immune responses. Based on recent evidence, molecularly targeted therapies increase NK cell-mediated tumor cell killing (Shimasaki et al., 2020). Several types of human cancers have been reported to contain mast cells, including malignant melanoma, breast, and colorectal cancers, which have a poor prognosis with mast cell infiltration (Komi and Redegeld, 2020). Persistence and antitumor immunity of memory T cells, including effector memory T cells (Tem) and central memory T cells (Tcm), have been demonstrated in several studies and may be used as a biomarker of immune responses against some cancers (Liu et al., 2020).

To better understand the possible mechanisms of KCNQ1OT1/hsa-miR-378a-3p/RBMS1, we examined the association between miR-378a-3p and the immune infiltrate in GC. Interestingly, miR-378a-3p showed the opposite results to RBMS1 during immune cell infiltration. Specifically, miR-378a-3p had a negative correlation with NK cells, Mast cells, Central Memory T cells, and Macrophage-related immune response. KCNQ1OT1 was also found to be negatively associated with NK CD56dim cells, neutrophils, Th1 cells, DC, and Macrophages. Previous studies have shown that CD56dim NK cells gradually decreased with disease progression in GC. Namely, GC severity is negatively correlated with the rate of CD56dim NK cells (Izawa et al., 2011).

Mutations, which are the cause of genetic variation, influence evolution. In addition to genetic mutations, changes in DNA methylation and sCNA play important roles in the transcriptional regulation of gene expression. However, no studies have comprehensively analyzed the associations between RBMS1 mutation, methylation, sCNA, and immune infiltration in GC. Our study revealed that RBMS1 has a higher mutation rate in GC than most cancers. Further, CD4^+^ T cell infiltration was found to be higher in GC patients with mutated RBMS1, while macrophage infiltration was lower in mutated RBMS1. Previous studies specifically revealed the importance of sCNA in tumorigenesis and tumor progression (Klijn et al., 2010). Moreover, high sCNA was associated with significantly increased ratios of anti-inflammatory cells, and lower ratios of CD8^+^ T cell compared with low sCNA level in GC. This correlation might be due to the progression of GC; increased levels were found in some immune cells that exhibit anti-inflammatory roles, such as macrophage/monocytes and neutrophils, while CD8^+^ T cell decreases gradually due to irreversible cell exhaustion. These findings suggest that a high mutation rate and high sCNA level of RBMS1 may promote immune infiltration and an immunosuppressive microenvironment and impact the prognosis of patients with GC.

This study had some limitations. First, an integrated analysis of the GC tissues is required to comprehensively verify how KCNQ1OT1/miR-378a-3p/RBMS1 is involved in the development of GC, which was not performed in the current study. Second, to align with the reported standards of prognostic models, further validation with larger patient datasets is needed to better estimate the accuracy of this model’s predictions in diverse patient populations. Finally, we expect to further validate the role of KCNQ1OT1/miR-378a-3p/RBMS1 axis in gastric cancer in the next study owing to the current experimental constraints.

These data highlight the contribution of multiple molecular pathways and biomarkers to GC, which is consistent with our current understanding of the disease’s pathophysiology. The results of this study provide new insights into the underlying molecular mechanisms of GC and reveal the KCNQ1OT1/miR-378a-3p/RBMS1 axis as a significant prognostic factor and therapeutic target for GC.

## Conclusion

In conclusion, this study aimed to identify the biological functions and pathways associated with the progression of GC through a comprehensive bioinformatics analysis and explore the molecular mechanisms underlying its progression.

Herein, a multigene prognostic model was constructed and the KCNQ1OT1/miR-378a-3p/RBMS1 axis was identified as an important prognostic factor and therapeutic target for GC, which may provide more insights into the correlation between lncRNA-miRNA-mRNA expression levels. However, the results of the current study should be validated through further molecular experiments.

## Data Availability

The original contributions presented in the study are included in the article/Supplementary Material, further inquiries can be directed to the corresponding authors.
