# Supplementary material for: Identification of the KCNQ1OT1/ miR-378a-3p/ RBMS1 Axis as a Novel Prognostic Biomarker Associated With Immune Cell Infiltration in Gastric Cancer
Source: Front Genet. 2022 Jul 14;13:928754. doi: 10.3389/fgene.2022.928754 (PMC9330051; doi:10.3389/fgene.2022.928754)
Supplement: Supplementary file 1 [file Table1.DOCX]

**Supplementary Table 1 |** Characteristics of patients in the training set and test set

| Characteristic | GSE84437  training set | GSE84437  test set | P value |
| --- | --- | --- | --- |
| n | 213 | 213 |  |
| Gender, n (%) |  |  | 1 |
| male | 145 (68.08%) | 146 (68.54%) |  |
| female | 68 (31.92%) | 67 (31.46%) |  |
| T stage, n (%) |  |  | 0.76 |
| T0_T2 | 187 (87.79%) | 190 (89.2%) |  |
| T3_T4 | 26 (12.21%) | 23 (10.8%) |  |
| N stage, n (%) |  |  | 0.63 |
| N0 | 181 (84.98%) | 165 (77.46%) |  |
| N1_N3 | 32 (15.02%) | 48 (22.54%) |  |
| OS status, n (%) |  |  | 0.63 |
| Death | 115 (53.99%) | 109 (51.17%) |  |
| Alive | 98 (46.01%) | 104 (48.83%) |  |
| OS_time (mean, sd) | 73.75 (47.87) | 68.95 (45.74) |  |
| Age (mean, sd) | 59.63 (12.06) | 60.28 (11.17) | 0.8 |
