# Supplementary figures and images for: Identification of the KCNQ1OT1/ miR-378a-3p/ RBMS1 Axis as a Novel Prognostic Biomarker Associated With Immune Cell Infiltration in Gastric Cancer
Source: Front Genet. 2022 Jul 14;13:928754. doi: 10.3389/fgene.2022.928754 (PMC9330051; doi:10.3389/fgene.2022.928754)

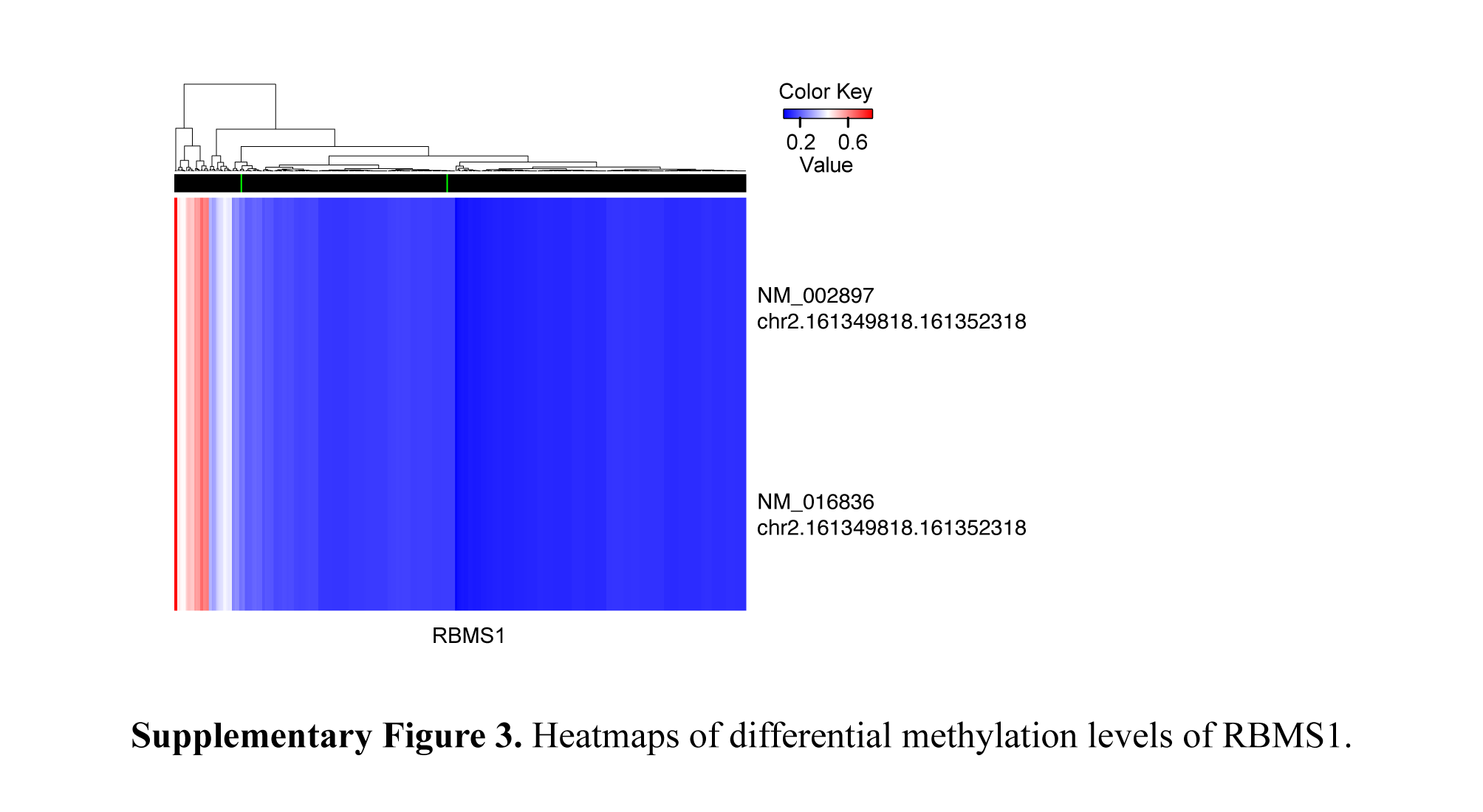

Supplement: Supplementary file 2 [file Image3.TIF]

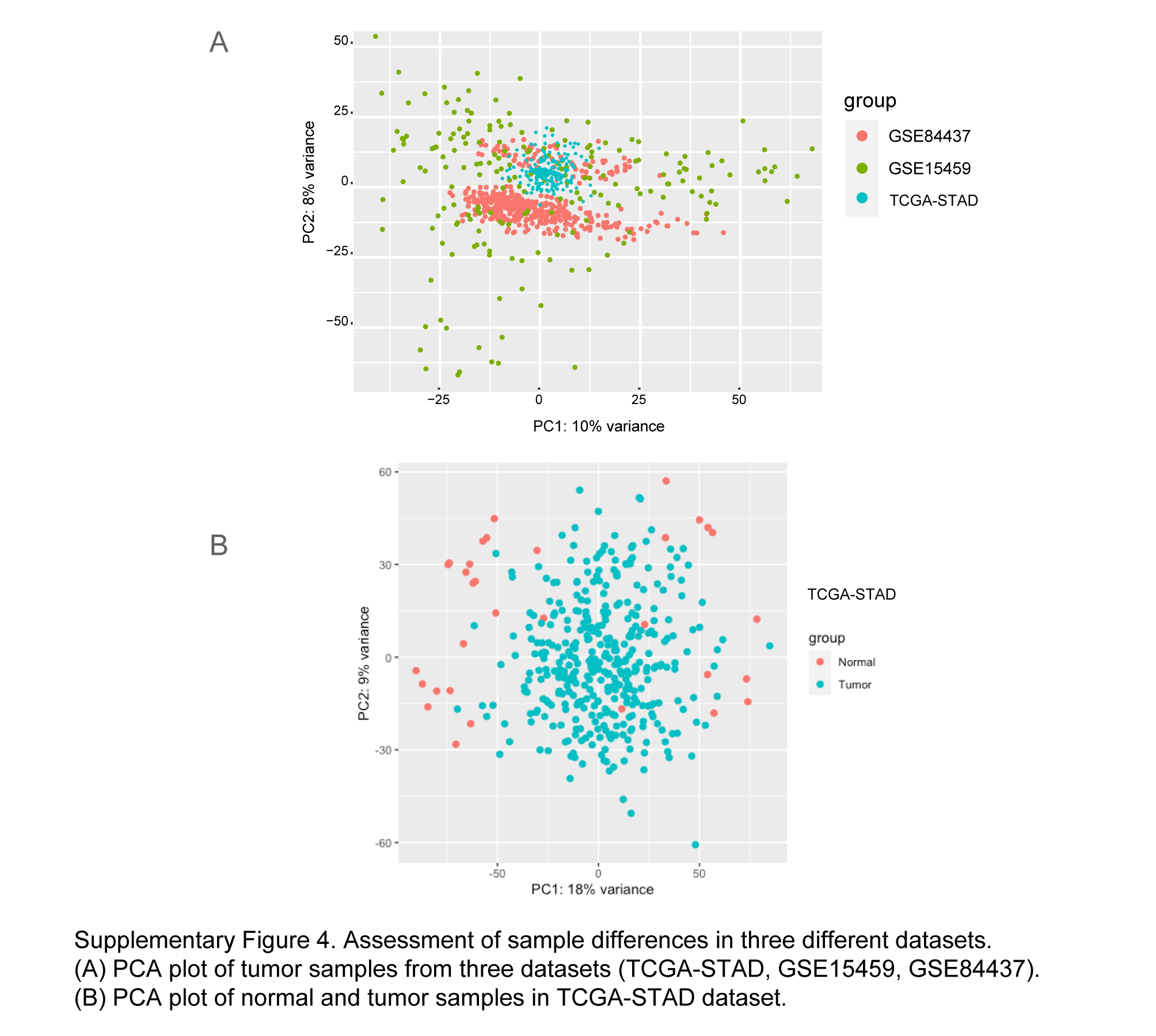

Supplement: Supplementary file 3 [file Image4.TIF]

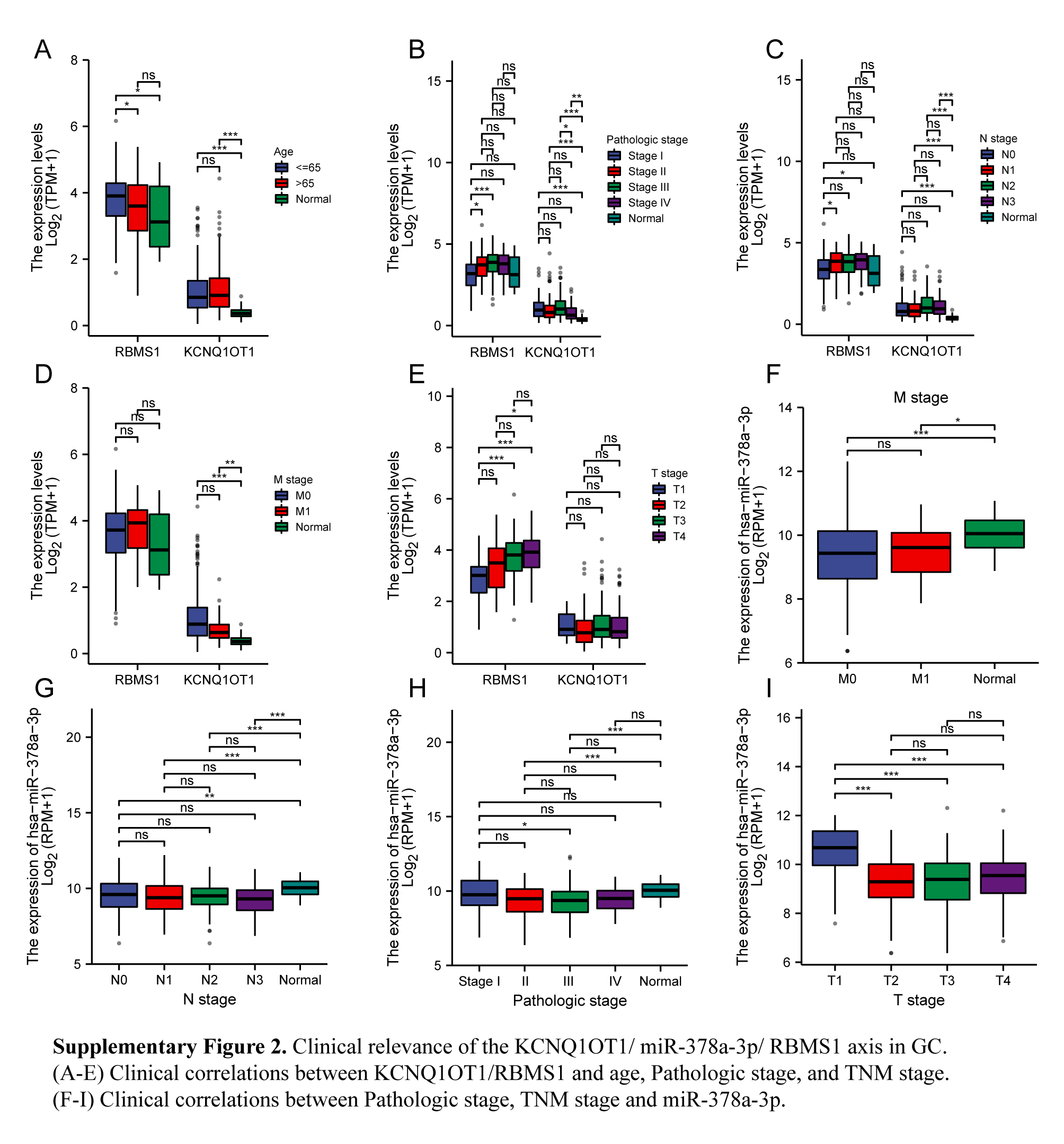

Supplement: Supplementary file 4 [file Image2.TIF]

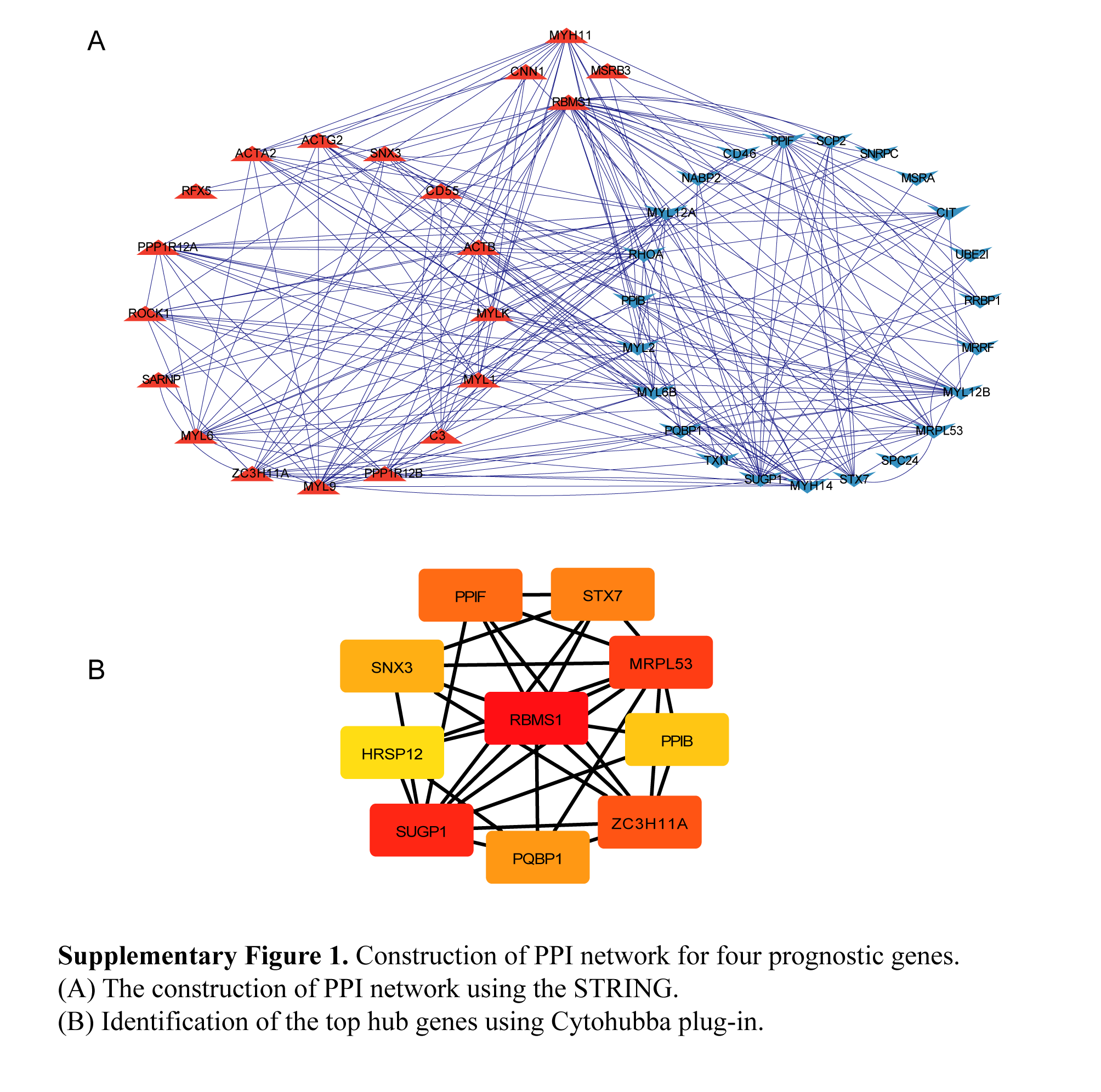

Supplement: Supplementary file 5 [file Image1.TIF]

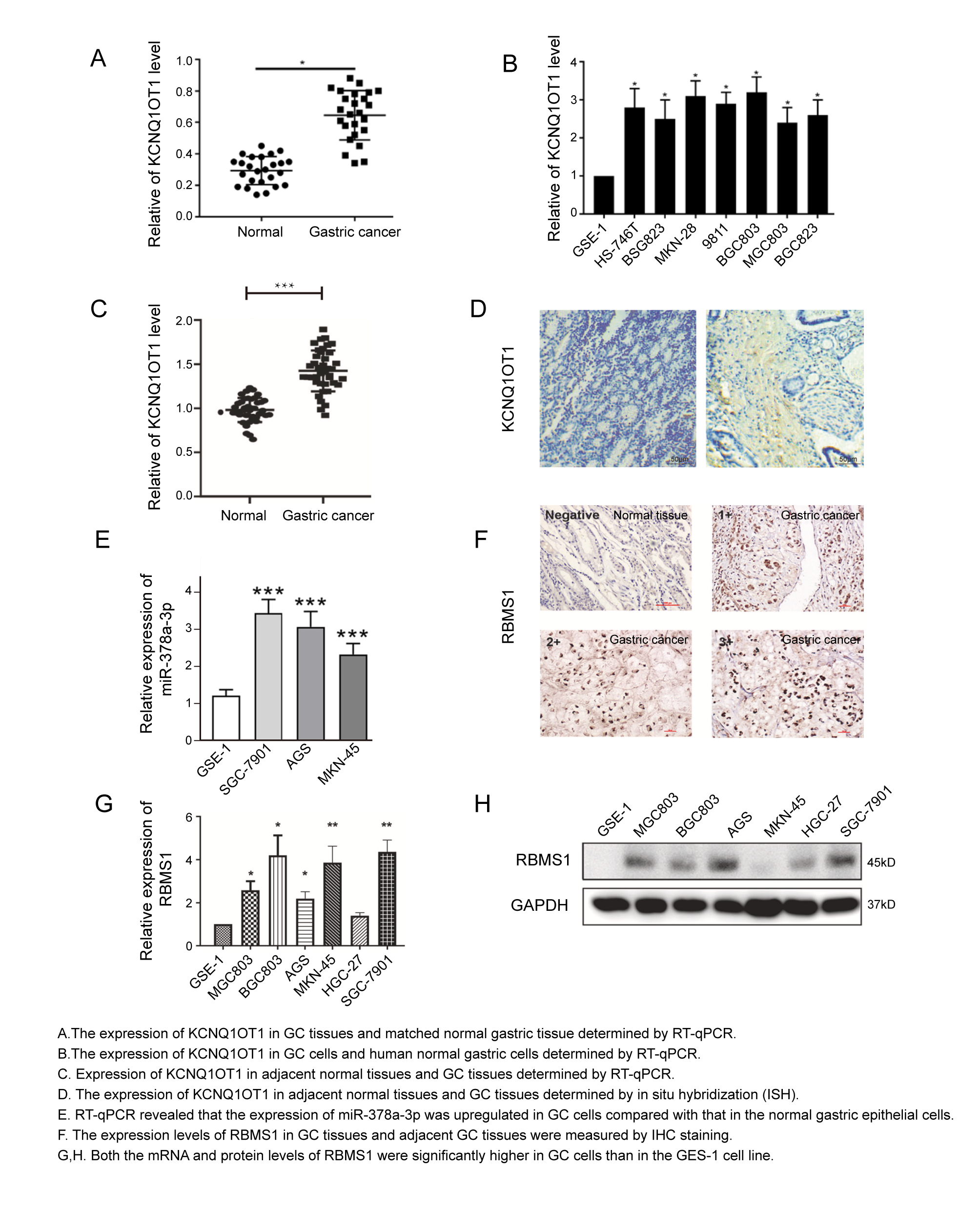

Supplement: Supplementary file 8 [file Image5.TIF]
