# Supplementary material for: Identification of the KCNQ1OT1/ miR-378a-3p/ RBMS1 Axis as a Novel Prognostic Biomarker Associated With Immune Cell Infiltration in Gastric Cancer
Source: Front Genet. 2022 Jul 14;13:928754. doi: 10.3389/fgene.2022.928754 (PMC9330051; doi:10.3389/fgene.2022.928754)
Supplement: Supplementary file 6 [file Table2.DOCX]

**Supplementary Table 2 |** Characteristics of patients in the GSE15459 dataset

| Characteristic | GSE15459 validation set |
| --- | --- |
| Gender, n (%) |  |
| Male  Female | 116 (64%)  66 (36%) |
| TNM, n (%) |  |
| Stage | 31 (17%) |
| Stage I | 28 (15%) |
| Stage III | 66 (36%) |
| Stage IV | 57 (31%) |
| OS status, n (%) |  |
| Alive | 87 (48%) |
| Death | 95 (52%) |
| OS time, median (IQR)  Age, median (IQR) | 20.3 (9.89, 63.66)  66.45 (57.05, 73) |
