# Supplementary material for: Identification of the KCNQ1OT1/ miR-378a-3p/ RBMS1 Axis as a Novel Prognostic Biomarker Associated With Immune Cell Infiltration in Gastric Cancer
Source: Front Genet. 2022 Jul 14;13:928754. doi: 10.3389/fgene.2022.928754 (PMC9330051; doi:10.3389/fgene.2022.928754)
Supplement: Supplementary file 7 [file Table3.DOCX]

**Supplementary Table 3 |** Characteristics of patients in the TCGA_STAD

| Characteristic | TCGA_STAD validation set |
| --- | --- |
| Gender, n (%) |  |
| Male  Female | 224 (64%)  124 (36%) |
| T stage, n (%) |  |
| T0_T2 | 64 (18%) |
| T3_T4 | 284 (82%) |
| N stage, n (%) |  |
| N0 | 100 (29%) |
| N1_N3 | 248 (71%) |
| M stage, n (%) |  |
| M0 | 325 (93%) |
| M1 | 23 (7%) |
| OS status, n (%) |  |
| Alive | 205 (59%) |
| Death | 143 (41%) |
| OS time, median (IQR) | 16.2 (9.75, 27.56) |
| Age, median (IQR) | 67 (58, 73) |
